# Determinants of depression in patients with comorbid depression following cardiac rehabilitation

**DOI:** 10.1136/openhrt-2018-000973

**Published:** 2019-04-09

**Authors:** Serdar Sever, Alexander Stephen Harrison, Su Golder, Patrick Doherty

**Affiliations:** Health Sciences, University of York, York, UK

**Keywords:** cardiac rehabilitation, depression, history of depression, comorbid depression, cardiovascular disease

## Abstract

**Background:**

A prior history of depression, at the point patients start cardiac rehabilitation (CR), is associated with poor outcomes; however, little is known about which factors play a part in determining the extent of benefit following CR. Therefore, we aim to identify and evaluate determinants of CR depression outcomes in patients with comorbid depression.

**Methods:**

An observational study of routine practice using the British Heart Foundation National Audit of Cardiac Rehabilitation data between April 2012 and March 2017. Baseline characteristics were examined with independent samples t-test and χ^2^ test. A binary logistic regression was used to predict change in depression outcome following CR.

**Results:**

The analysis included 2715 CR participants with depression history. The determinants of Hospital Anxiety and Depression Scale (HADS) depression measurement post-CR were higher total number of comorbidities (OR 0.914, 95% CI 0.854 to 0.979), a higher HADS anxiety score (OR 0.883, 95% CI 0.851 to 0.917), physical inactivity (OR 0.707, 95% CI 0.514 to 0.971), not-smoking at baseline (OR 1.774, 95% CI 1.086 to 2.898) and male gender (OR 0.721, 95% CI 0.523 to 0.992).

**Conclusion:**

Baseline characteristics of patients with comorbid depression such as higher anxiety, higher total number of comorbidities, smoking, physical inactivity and male gender were predictors of their depression levels following CR. CR programmes need to be aware of comorbid depression and these related patient characteristics associated with better CR outcomes.

**Key questions**

**What is already known about this subject?**

- A prior history of depression is associated with poor cardiac rehabilitation outcomes; however, little is known about which factors play a part in determining the extent of benefit after cardiac rehabilitation in patients with history of depression.

**What does this study add?**

- This study is the first to identify and evaluate the factors that determine cardiac rehabilitation depression outcomes in patients with prior history of depression and inform future cardiac rehabilitation practice.

**How might this impact on clinical practice?**

- Cardiac rehabilitation programmes need to be aware of patients with history of depression and their modifiable baseline patient characteristics such as anxiety, smoking and physical inactivity associated with outcomes to ensure patients with history of depression gain the most from CR programmes and improve their depression levels after cardiac rehabilitation.

## Introduction

Cardiovascular disease (CVD) constitutes the highest mortality rate among all causes of death and is responsible for 17.9 million deaths around the world in 2016^1^ and approximately 152 500 deaths in the UK in 2016.^2^ Additionally, 83.5 million people in European Society of Cardiology member countries continue to live with CVD.^3^ Due to increased survival rates in CVD and an ageing population, there has been an increase in the number of comorbidities present with CVD which further complicates the management of a patient’s condition and service delivery options.^4^ Depression is reported to be a prevalent comorbidity in cardiac patients; the prevalence of depression ranges from 15%–20% to nearly 50% depending on the assessment measurement ranging from structured interviews applied for clinical diagnosis to self-answered survey questions, respectively.^5^ Furthermore, several systematic reviews have shown that depression is an independent risk factor for cardiac mortality^6^ as well as all-cause mortality.^7 8^ In addition, the evidence suggests that when depression and CVD present together, they have a significant influence on both medical costs and service delivery.^9^

Cardiac rehabilitation (CR) is a multicomponent intervention that incorporates secondary prevention for CVD.^10 11^ Recent systematic reviews have shown that CR is an effective intervention^12 13^ and reduces depressive symptoms.^14^ Two cohort studies have also shown the impact of CR on reducing depression levels in patients with CVD^15 16^ in accordance with a literature review.^17^ The management of psychosocial health has been promoted in recent guidelines and the importance of multicomponent CR programmes highlighted.^18 19^ Thus, a measure of psychosocial health status is required to assess and manage depression symptoms among CR participants. Hospital Anxiety and Depression Scale (HADS) is used in the UK context and applied before and after CR to tailor interventions considering patient’s needs.^20^ HADS is a valid measurement of depressive symptoms in CVD populations in the UK and also in other countries.^21^ Most prior studies have focused on depressive symptoms associated with the acute event, and there is very little evidence on the influence of prior history of depression.^8^ In addition, some studies have shown that patients with CVD with comorbid depression prior to their heart event were at increased risk in terms of mortality and poor cardiac prognosis.^22 23^ Therefore, we aim to identify and evaluate sociodemographic and clinical characteristics associated with depression outcome in patients with historic comorbid depression following CR.

## Methods

An observational study of routine practice across England, Wales and Northern Ireland using the British Heart Foundation National Audit of Cardiac Rehabilitation (NACR) is employed. The checklist ‘Strengthening the reporting of observational studies in epidemiology’ (STROBE) is followed to report this study.^24^

### Data collection

NACR data are used to perform the analysis. The NACR is responsible for monitoring the UK CR services and improving the quality of these programmes. The data regarding patient’s demographics and clinical characteristics are collected via CR programmes and entered by the approved member of clinical teams to a secure online platform, NHS digital, under the section 251 approval. Baseline and post-CR assessments are routinely conducted and then data are link-anonymised and transferred to NACR located at the University of York. A total of 224 CR services provide data which constitutes 74% of all programmes in the UK.^20^ The data include patients who participated in CR along with details of their initiating event, treatment, demographics, risk factors, medication and outcomes.

### Participants

The data of the last 5 years based on NACR—1 April 2012 to 31 March 2017—were extracted and analysed. CR guidelines have recommended the inclusion of patients with myocardial infarction and heart failure and those who receive treatment of percutaneous coronary intervention and coronary artery bypass graft,^25^ which constituted the study population. All the eligible patients (N=2715) with comorbid depression who had pre-HADS and post-HADS assessments in CR were selected as participants during the study period.

### Measures

#### Comorbid depression

Comorbid depression is operationalise here as a medical history of depression in the NACR data which is confirmed by CR practitioners with case note review and also by patients in terms of whether they have ever been diagnosed or treated by a doctor for depression. Therefore, comorbid depression is defined in the paper as depression history prior to heart event.

#### Hospital Anxiety and Depression Scale

The HADS is a self-answered screening tool to measure depressive symptoms which is widely used in many clinical populations. In a recent CR guideline, psychosocial health assessments, using HADS, are recommended to be applied before and after CR to tailor the interventions to cardiac patients.^18^ The HADS is a validated measure licensed to NACR for the assessment of anxiety and depression symptoms and is recommended for use with patients with CVD.^21^ The HADS is scored between 0 and 21 where a higher score is associated with worse symptoms.^26^ The clinical cut-off point of 8 is used to categorise patients to low levels of depression (<8) and higher depression groups (≥8).^21^ Statistical analyses were conducted comparing patients with HADS <8 and HADS ≥8 in a subgroup of patients with comorbid depression. Baseline HADS anxiety scores, which are routinely reported as part of HADS, were assessed to see whether it predicts depression outcomes following CR.

#### Total number of comorbidities

Total number of comorbidities are defined as the sum of the number of comorbidities that the patients had including hypertension, hypercholesterolaemia, diabetes, angina, arthritis, osteoporosis, asthma, chronic bronchitis and others. There are 19 different comorbidities in the NACR data.

#### Physical activity

Physical activity was measured by the question of “Do you take regular moderate physical activity of at least 30 min duration on average 5 times a week?” which requires an answer of ‘yes’ or ‘no’. This gives the proportion of moderate physical activity. Moderate activity is explained in the questionnaire as anything that takes as much effort as brisk walking or housework, carrying a light bag on level ground, mowing the lawn, painting and decorating, sports like easy swimming, easy cycling or hobbies such as ballroom dancing.

#### Smoking

Baseline smoking measurements are categorised as to whether a patient smokes or not. If patients never smoked, ex-smoker or stopped smoking since the heart event, they are categorised as non-smoking at baseline and if they were currently smoking categorised as smoking. Self-reported smoking is a valid measurement and shows similar results with biochemical smoking assessments.^27^

#### Weight

Weight was measured in kilograms. It is measured by CR programmes.

#### Marital status

Marital status was determined by whether patients have a partner or not at baseline. Patients who are married or have permanent partnership were categorised into partnered and the ones who are single, divorced, widowed and separated categorised into not-partnered or single.

### Statistical analysis

SPSS V.24 is used to conduct the data analysis. Patients with comorbidity depression who have valid pre-HADS and post-HADS assessments constituted the study population. Statistical significance was set to 5%. Summary statistics were presented as mean and SD unless otherwise stated. Baseline characteristic for the variables of age, number of comorbidities, weight, HADS anxiety measurement, gender, physical activity, smoking and marital status were investigated. Using these variables, defined from the literature and baseline assessments, binary logistic regression were conducted to see which of these variables predict change in HADS depressive symptoms. We investigated which baseline characteristics predict moving to low levels of depressive symptoms in comparison with remaining with higher depressive symptoms after CR.

### Ethics

Approved members of clinical teams enter NACR data to NHS digital which is a secure online environment that meets General Data Protection Regulation (GDPR) requirements. NHS digital has the approval to collect the patient-identifiable data which is then link anonymised and sent electronically to NACR for the quality validation of the data. As part of NHS digital contract, data on pre-CR and post-CR assessments and characteristics of patients are collected for the purpose of audit and service improvement. On this basis no separate ethical approval was required.

## Results

**Figure 1:**
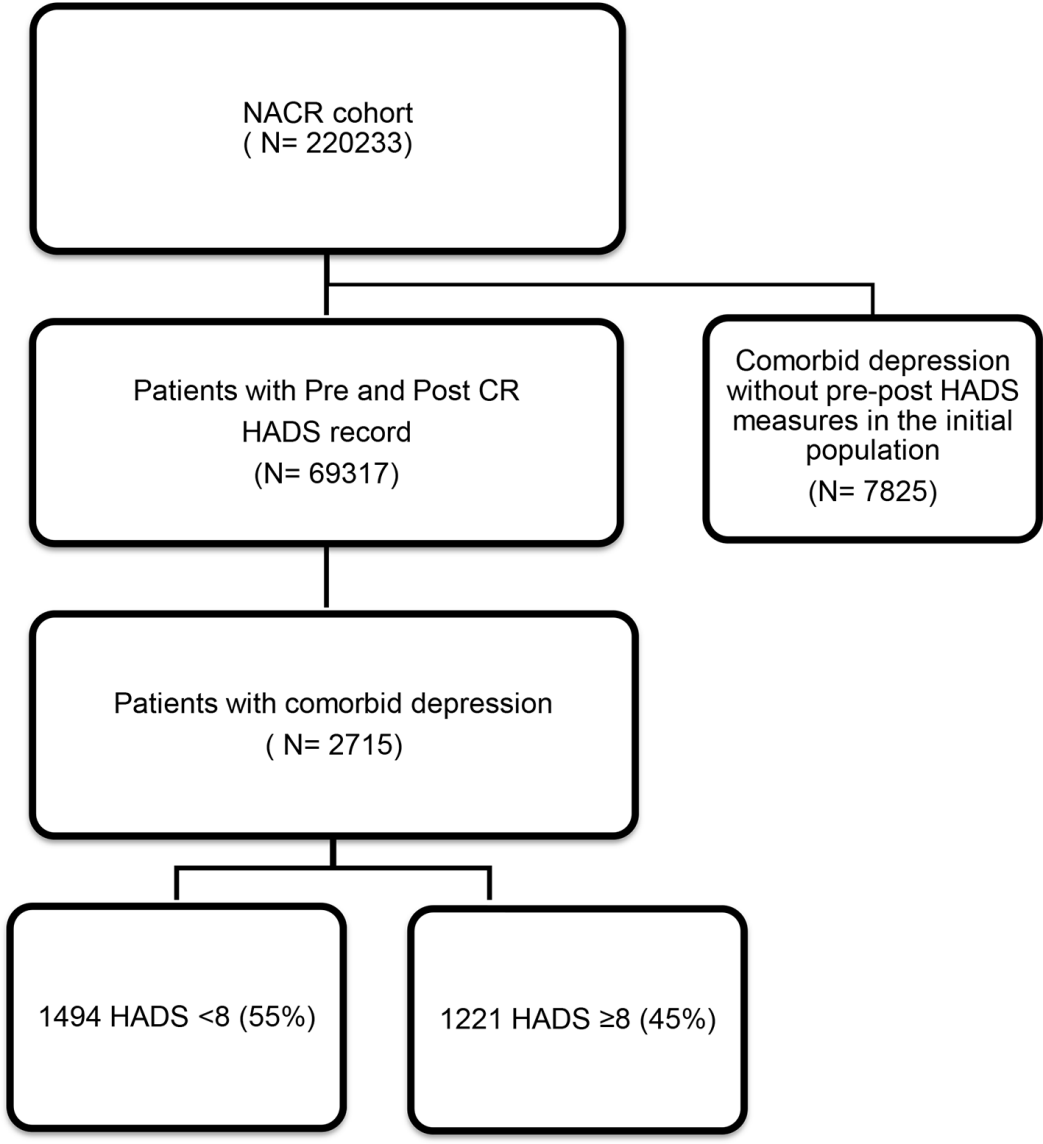
Flow diagram for study sampling. CR, cardiac rehabilitation; HADS, Hospital Anxiety and Depression Scale; NACR, National Audit of Cardiac Rehabilitation.

The study population was 2715 participants with comorbid depression who have completed CR with valid pre-HADS and post-HADS assessments. Of these 2715 participants, 55% had low level of depressive symptoms (HADS <8) and 45% had a higher level of depressive symptoms (HADS ≥8). The flow diagram in figure 1 shows the total population in the time period and sample size of our study. At baseline, participants with higher depressive symptoms group were younger, had higher number of comorbidities, increased weight, higher anxiety, were less physically active, more likely to be smoker and single. Baseline characteristics of participants in the context of HADS are presented in table 1.

**Table 1:** Baseline characteristics for high and low HADS depression groups

Variables	HADS <8 group (n=1494)	HADS ≥8 group (n=1221)	P value	Effect size
	Mean±SD	Mean±SD		
Age	63.49±10.50	60.77±10.53	<0.001	0.26
Total comorbidities	4.39±2.10	4.90±2.19	<0.001	0.24
Weight	83.62±17.04	85.56±19.08	0.001	0.11
HADS anxiety score measurement	6.43±3.80	11.60±4.05	<0.001	1.32
Gender male %	66.4	66.4	0.99	0
150 min physical activity a week (yes) %	43.6	27.5	<0.001	0.17
Smoking (yes) %	7.7	12.7	<0.001	0.08
Partnered %	71.5	63.6	<0.001	0.08

HADS, Hospital Anxiety and Depression Scale.

A binominal logistic regression was performed to ascertain the impact of age, gender, weight, total number of comorbidities, HADS anxiety measurement, physical activity, smoking and marital status on the likelihood that participants move to low levels of depression group over the ones who remain in the higher levels of depression symptoms group after CR. The logistic regression model was statistically significant, χ^2^(8)=72.193, p<0.001. The model correctly classified 63.3% of the cases. Hosmer and Lemeshow test shows that the model was a good fit (p=0.288). Of the eight predictor variables, five were statistically significant: total number of comorbidities, HADS anxiety score measurement, physical activity, smoking and gender (as shown in table 2).

**Table 2:** Coefficients of the model predicting change in depression whether a patient has moved to low levels of depressive symptoms group after CR

Variable	B	SE	P value	Lower 95% CI	OR	Upper 95% CI
Age	−0.002	0.008	0.765	0.983	0.998	1.013
Total no of comorbidities	−0.090	0.035	0.010	0.854	0.914	0.979
Weight	−0.004	0.004	0.363	0.988	0.996	1.004
HADS anxiety score measurement	−0.124	0.019	<0.001	0.851	0.883	0.917
150 min a week physical activity (no)	−0.347	0.162	0.033	0.514	0.707	0.971
Smoking (no)	0.573	0.250	0.022	1.086	1.774	2.898
Gender (male)	−0.328	0.163	0.045	0.523	0.721	0.992
Marital status (single)	−0.088	0.155	0.570	0.677	0.916	1.240

B, regression coefficient;CR, cardiac rehabilitation.

Patients with higher number of comorbidities had 0.914 times lower odds of residing in low levels of depressive symptoms after CR (OR 0.914, 95% CI 0.854 to 0.979). Increased HADS anxiety score measurement was associated with a decreased likelihood of moving to low levels of depressive symptoms group after CR (OR 0.883, 95% CI 0.851 to 0.917). Physical inactivity and male gender were also associated with reduced odds of becoming low depression level category (with OR 0.707, 95% CI 0.514 to 0.971 and OR 0.721, 95% CI 0.523 to 0.992, respectively), whereas non-smoking was associated with increased likelihood of having low HADS range after CR (OR 1.774, 95% CI 1.086 to 2.898).

## Discussion

Prior studies have tackled certain elements of the association between depressive symptoms and lifestyle risk factors along with patient demographics. However, the impact of comorbid depression was not clear. Therefore, in the current study, the influence of comorbid depression in patients with CVD has been evaluated to a greater extent through a robust approach. The results of our study has shown that baseline characteristics of participants with comorbid depression determine outcome following CR. These characteristics such as having higher total number of comorbidities, higher anxiety scores, physical inactivity, smoking and male gender were significant predictors of depression outcomes after CR. However, age, weight and marital status were not able to determine depressive symptoms following CR in patients with a history of comorbid depression.

Our findings which are unique by virtue of being based on routine clinical practice data support the previous cohort and trial studies which have shown the association with physical inactivity^28 29^ and smoking,^30 31^ with higher depressive symptoms. One additional finding of note was that baseline anxiety scores had an impact on depression outcomes (p<0.001, OR 0.883, 95% CI 0.851 to 0.917). One recent study has shown that anxiety and depression are, when presented together, associated with increased mortality rates after CR (HR 2.41, p=0.04).^32^ Our study shows that baseline anxiety scores of patients with history of depression predicts outcome after CR. Patients with history of depression are also more likely to remain depressed when they have high anxiety scores at baseline. Therefore, participants’ anxiety levels at baseline may be an important CR intervention focus for reducing mortality rates in patients with comorbid depression history. Further studies assessing the effectiveness of such interventions would be valuable.

Higher total number of comorbidities was also a significant predictor of depressive symptoms in our study (OR 0.914, 95% CI 0.854 to 0.979), although one other study has stated the opposite in the general population.^33^ However, a recent study has shown that increased number of comorbidities limit cardiac patients’ physical activity and correspondingly reduce their quality of life^34^; perhaps this may mediate having higher depressive symptoms as a consequence. In addition, this association of higher number of comorbidities with higher depressive symptoms also becomes a salient finding considering that 19 different comorbidities were included in the NACR data such as hypertension, hypercholesterolaemia, diabetes, angina, arthritis, osteoporosis, asthma, chronic bronchitis and others. Besides, the findings of another study were also in line with this which has shown the association of comorbidities with depressive symptoms in the general population of older adults in western European countries.^35^

A recent meta-analysis which included 49 prospective studies with a sample around 267 000 individuals has shown that physical activity had a protective effect on incident depression regardless of geographical region in the general population (OR 0.83, 95% CI 0.79 to 0.88).^36^ However, our study adds, given prior depression history, cardiac patients who are less physically active were 30% less likely to move to low levels of depressive symptoms group after CR (OR 0.707, 95% CI 0.514 to 0.971). Another clinically important finding was that, as an important lifestyle risk factor, smoking was influential on CR outcomes. The present study has shown that participants who are non-smokers at baseline were 77% more likely to move to low levels of depression symptoms group after CR (OR 1.774, 95% CI 1.086 to 2.898). The results of a recent systematic review has also supported the association of smoking with depressive symptoms in the general population.^37^ A total of 148 cohort studies included in this review and a third of included studies explored the association between smoking status with later depression. Of these studies, 73% of them have found evidence to support this association. Our findings have supported this association in patients with CVD who participated in CR programmes in routine CR practice.

One finding was that age was not a significant determinant of depression (OR 0.998, 95% CI 0.983 to 1.013) as it was in a prior study concluding that patients with higher depressive symptoms were younger.^38^ Although this relationship was seen at baseline, age was not able to predict outcome after adjusting for other covariates in our model. Unlike prior studies, weight did not have a potential impact on depressive symptoms.^39^ A possible explanation for this might be that we have accounted for total number of comorbidities which could be predictive of weight change.^40^ Furthermore, marital status in patients was not able to predict depressive symptoms (OR 0.916, 95% CI 0.677 to 1.240) which differs from findings observed in the general population.^41^ Although this association was observed at baseline, adjusting for other covariates it no longer reached statistical significance in the final model. A recent prospective study in Finland found an association between increased marriage dissatisfaction and risk of sudden cardiac death among men (HR 1.90, 95% CI 1.09 to 3.32).^42^ The NACR does not have information of the satisfaction of patients on their marriage; however, this could be an important factor for further studies to consider rather than looking at whether patients have a partner or not. A finding was that men were less likely to gain improvement in their depressive symptoms than women in our comorbid depression sample (OR 0.721, 95% CI 0.523 to 0.992). This result differs from a previous study.^43^ Although we have included more women in our study (33.6%), their mean age was lower, by 3 years, which makes it hard to explain this result. However, proportion of male smokers was higher than female smokers in our sample which may have an impact on the result as smoking is associated with higher levels of depression.

An important finding for clinical practice was that 60%–65% (N=5110) of patients in routine practice with comorbid depression did not have recorded pre-CR and post-CR HADS measurements. Clinical guidance across the globe recommend that psychosocial health status is assessed before and after rehabilitation.^18 19^ As our study shows, HADS measurement, in patients with prior history of depression, informs patient outcomes. Therefore, CR programmes need to be aware of this and aim to carry out and record psychosocial assessment before and after CR. Our study is the first to establish this in the UK CR programmes.

### Limitations

One limitation is that our population is a subgroup of those with a pre-existing diagnosis of depression. This is because we wanted to test what are the characteristics of patients with comorbid depression determining CR outcome. However, when looking at our population, it is representative of all available patients with comorbid depression as patient demographics were similar between our sample and all available participants with comorbid depression (mean age was 62 compared with 61 and 33.6% women compared with 35%), and this proportion did not differ more than 5% for other variables. Although the sample was nationally representative of patients with comorbid depression around the UK, it is important to state that not all CR programmes in the UK provide a complete record of patients who completed CR. As many as 38% did not have follow-up assessment in NACR data which may have an impact on the representativeness of the sample.^20^ However, an observational study enabled us to understand what happens in the real world by analysing routinely collected data which is one of its main strengths. In addition, the data included patients with multi-comorbidities and more female participants than prior RCTs.^12^

## Conclusion

An initial objective of the project was to identify which factors determine the depressive symptoms in patients with comorbid depression in CR. Baseline characteristics of patients with comorbid depression such as higher anxiety, higher total number of comorbidities, smoking, physical inactivity and male gender were associated with their depression levels following CR. CR programmes need to be aware of this specific population of patients with comorbid depression and their characteristics to improve CR outcomes. CR programmes should target HADS measurements towards patients with comorbid depression and give importance to recording as part of routine practice. As this is the first study investigating history of depression in CR literature, further studies on the current topic are therefore recommended to build on our findings.
